# Subthalamic deep brain stimulation improves sleep and excessive sweating in Parkinson’s disease

**DOI:** 10.1038/s41531-020-00131-0

**Published:** 2020-10-14

**Authors:** Silje Bjerknes, Inger Marie Skogseid, Tuva Jin Hauge, Espen Dietrichs, Mathias Toft

**Affiliations:** 1 Department of Neurology, Oslo University Hospital, Oslo, Norway; 2 Institute of Clinical Medicine, University of Oslo, Oslo, Norway

**Keywords:** Parkinson's disease, Randomized controlled trials

## Abstract

Parkinson’s disease (PD) is a complex multisystem disorder with motor and non-motor symptoms (NMS). NMS may have an even greater impact on quality of life than motor symptoms. Subthalamic nucleus deep brain stimulation (STN-DBS) has been shown to improve motor fluctuations and quality of life, whereas the effects on different NMS have been less examined. Sleep disturbances and autonomic dysfunction are among the most prevalent NMS. We here report the efficacy of STN-DBS on sleep disturbances and autonomic dysfunction. In the parent trial, 60 patients were included in a single-center randomized prospective study, with MDS-UPDRS III and PDQ-39 as primary endpoints at 12 months of STN-DBS. Preplanned assessments at baseline and postoperatively at 3 and 12 months also included Parkinson’s Disease Sleep Scale (PDSS); Scopa-Aut; and MDS-UPDRS I, II, and IV. We found that STN-DBS had a significant and lasting positive effect on overall sleep quality, nocturnal motor symptoms and restlessness, and daytime dozing. Several aspects of autonomic dysfunction were also improved at 3 months postoperatively, although at 12 months only thermoregulation (sudomotor symptoms) remained significantly improved. We could not identify preoperative factors that predicted improvement in PDSS or Scopa-Aut. There was a close relationship between improved autonomic symptoms and improved quality of life after 1 year. NMS and especially sleep and autonomic dysfunction deserve more focus to improve patient outcomes further.

Parkinson’s disease (PD) is a common neurodegenerative disease diagnosed by the classical motor signs of bradykinesia, tremor, and/or rigidity^1^. However, it has been increasingly recognized that PD is a complex multisystem disorder with motor and non-motor symptoms (NMS)^2,3^. NMS can be divided into four domains: autonomic, sleep, neuropsychiatric, and sensory symptoms, including pain^4,5^. NMS have a significant impact on quality of life^6^, often even to a greater extent than motor symptoms^7–10^.

Treatment of PD aims at reducing symptoms, as no treatment that can modify disease progression has been established. Symptom reduction through treatment with levodopa and other dopaminergic agents is highly effective, but in later disease stages the management of motor and non-motor fluctuations can be challenging. When oral medical treatment proves to be insufficient in reducing tremor or motor fluctuations, deep brain stimulation (DBS) is an effective and well-established symptomatic treatment option^11–13^. The significance of NMS in the DBS-treated cohorts and the effect of DBS on these symptoms are reflected by several publications on this topic during the past years^14–16^.

NMS burden has been shown to increase by disease duration, age, and with more severe motor impairment^17^. The patients eligible for subthalamic nucleus (STN)-DBS typically are in an advanced stage of the disease when the NMS burden is high, but the reported effects of STN-DBS on NMS are somewhat conflicting in the literature. Some studies have shown that DBS is better than dopamine replacement therapy in reducing NMS^18^, others have shown only minor effects of STN-DBS compared to levodopa and that the pattern of NMS was similar in a non-operated PD reference population^19,20^. Improvements in NMS have, however, been shown in several studies^21–23^. The literature thus presents variable findings in this research field, which is impeded by the use of many different methods and questionnaires, including non-validated scales or questionnaires^24,25^.

Sleep problems have been reported in 60–98% of PD patients^26,27^, but autonomic NMS are also common (nocturia in 62% of patients, urinary urgency in 58%, hypersalivation in 48%)^28^. Despite the high frequency, such symptoms have been less investigated than the total NMS burden. To our knowledge, few long-term studies have investigated the effect of STN-DBS on sleep and dysautonomia.

In this study, we have evaluated the impact of bilateral STN-DBS on sleep problems and dysautonomia during the first year of chronic stimulation and explored their relationship to motor and quality-of-life outcomes.

## Results

In this study, 60 patients were included, operated, and examined preoperatively, with planned follow-up after 3 and 12 months of continuous STN-DBS^29^. There were no significant differences in Parkinson’s Disease Sleep Scale (PDSS) or Scopa-Aut (scales for outcomes in PD—autonomic) between the two randomization groups of the parent study (see “Methods” section), neither regarding improvement from baseline to the 12-month follow-up nor in the scores at the different time points. Thus, for the purpose of this paper, we analyzed the two groups as one sample. Median age at surgery was 62 (44–71) years, disease duration 11 (4–23) years, and median preoperative Movement Disorder Society revision of the Unified Parkinson’s Disease Rating Scale (MDS-UPDRS) III scores in the medication-off/-on states were 47/13 (range 23–78/1–45). Complete baseline characteristics are presented in Table 1.

**Table 1:** Baseline characteristics.

Gender (*n* (%))	
Male	45 (75)
Female	15 (25)
Age at surgery	62 (44–71)
Disease duration (years)	11 (4–23)
HAD	
Anxiety	4 (0–12)
Depression	3 (0–19)
Mattis Dementia Rating Scale	142 (131–144)
LEDD	1291 (428–2490)
MDS-UPDRS I	10.5 (1–25)
MDS-UPDRS II	16.0 (0–32)
MDS-UPDRS III	
Off	47 (23–78)
On	13 (1–45)
MDS-UPDRS IV	10.0 (0–16)
H&Y off (*n* (%))	
1	0
1.5	0
2	20 (33)
2.5	17 (28)
3	10 (17)
4	11 (18)
5	2 (3)
H&Y on (*n* (%))	
1	3 (5)
1.5	3 (5)
2	37 (62)
2.5	15 (25)
3	2 (3)
4	0
5	0

Values are medians (min–max). *N* = 60 except for HAD *n* = 58 and Mattis Dementia Rating Scale *n* = 50.

Three patients had surgical site infections with subsequent hardware explantation and discontinuation of neurostimulation (before the 3-month follow-up), and two patients were lost to 1-year follow-up. Significant improvements were found for MDS-UPDRS I–IV, Parkinson’s Disease Questionnaire-39 (PDQ-39), and levodopa equivalent daily dosage (LEDD)^30^ at both the 3- and 12-month evaluations, as presented in our previous publication^29^ (Table 2).

**Table 2:** Changes from preoperative to the 3- and 12-month follow-up for PDSS, Scopa-Aut, PDQ-39, MDS-UPDRS I–IV, and LEDD.

	Preoperative	3 months	12 months	*p*
PDSS, *n*	58	49	52	
PDSS total	93.8 (21.3)	110.0 (21.7)	107.3 (21.3)	<0.001^b^
Overall quality of night’s sleep (Q1)	5.0 (2.8)	6.5 (2.9)	6.2 (2.8)	<0.001^b^
Sleep onset and maintenance insomnia (Q2, Q3)	10.9 (4.1)	12.9 (4.8)	12.0 (4.4)	0.007
Nocturnal restlessness (Q4, Q5)	11.5 (5.2)	14.1 (4.9)	13.0 (5.9)	0.003^b^
Nocturnal psychosis (Q6, Q7)	16.8 (3.4)	17.5 (2.7)	16.4 (4.9)	0.120
Nocturia (Q8, Q9)	11.7 (3.8)	12.4 (4.2)	12.5 (4.0)	0.055
Nocturnal motor symptoms (Q10–Q13)	26.2 (8.9)	32.4 (6.0)	33.1 (6.4)	<0.001^b^
Sleep refreshment (Q14)	5.2 (3.0)	6.3 (3.0)	6.0 (3.1)	0.041
Daytime dozing (Q15)	6.5 (3.3)	8.0 (2.4)	7.6 (3.2)	<0.001^b^
Scopa-Aut, *n*	58	54	53	
Scopa-Aut total	16.9 (8.2)	13.0 (6.8)	15.2 (8.4)	<0.001^b^
Gastrointestinal (Q1–7)	5.0 (3.3)	4.2 (2.9)	4.8 (3.6)	0.059
Urinary (Q8–13)	5.8 (3.7)	4.4 (2.7)	5.2 (3.7)	0.054
Cardiovascular (Q14–16)	0.8 (1.1)	0.6 (0.9)	0.6 (1.0)	0.172
Thermoregulatory (Q17–21)	3.2 (2.5)	1.8 (1.8)	2.2 (2.3)	<0.001^b^
Pupillomotor (Q19)	0.7 (0.8)	0.5 (0.6)	0.7 (0.9)	0.059
Sexual (Q22–25)	1.4 (1.8)	1.5 (1.8)	1.6 (2.0)	0.622
PDQ, *n*	59	51	53	
PDQ-39	27 (12)	18 (13)	20 (15)	<0.001
MDS-UPDRS, *n*	60	56	55	
MDS-UPDRS I	11.3 (6.1)	8.7 (5.8)	9.0 (5.7)	<0.001
MDS-UPDRS II	16.8 (7.3)	10.9 (6.5)	11.5 (6.7)	<0.001
MDS-UPDRS III off	49 (13)	19 (10)	20 (9)	<0.001
MDS-UPDRS III on	14 (9)	12 (7)	12 (7)	<0.001
MDS-UPDRS IV^a^	9.6 (3.5)	2.5 (3.0)	2.6 (3.7)	<0.001
LEDD	1301 (441)	689 (373)	639 (328)	<0.001

Mean (SD) are shown for all patients who had complete scores at each time point.

Significance of changes (*p* value presented) was tested with one-way repeated-measures ANOVA in subjects with values at all three time points; for PDSS *n* = 44 and for Scopa-Aut *n* = 51.

^a^Friedman test.

^b^Significant also after Bonferroni correction (for PDSS significance level *p* < 0.006, for Scopa-Aut significance level *p* < 0.007).

### Changes of PDSS and Scopa-Aut from baseline to 3- and 12-month follow-up

Mean PDSS total score improved significantly from 93.8 (±21.3) at baseline to 110.0 (±21.7) at 3 months and 107.3 (±21.3) at 12 months of STN-DBS. Changes for each domain are shown in Table 2. Overall sleep quality, nocturnal restlessness, nocturnal motor symptoms, and daytime dozing were all significantly improved. For sleep onset and maintenance insomnia, improvements were just above significance level after Bonferroni correction.

**Fig. 1:**
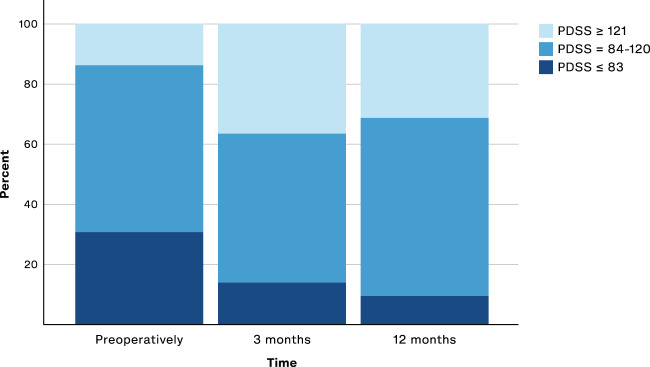
PDSS severity distribution. Columns show the percentage of patients with PDSS ≤83 (indicating severe sleep abnormality), PDSS 84–120 (indicating sleep disturbances), and PDSS ≥121 (indicating no sleep disturbances), preoperatively (*n* = 58) versus after 3 (*n* = 49) and 12 months (*n* = 51) of STN-DBS. McNemar’s test preoperative to 3 months (*p* = 0.001) and preoperative to 12 months (*p* = 0.005).

The proportion of patients with severe sleep abnormalities (PDSS score ≤ 83 points)^31^ were reduced from 31% preoperatively to 14% at 3 months (McNemar’s test, *p* = 0.001) and to 12% at 12 months (*p* = 0.005), see Fig. 1.

Scopa-Aut total score improved significantly overall over the three time points (repeated-measures analysis of variance (ANOVA)), see Table 2. Preoperative mean (±SD) score was reduced from 16.9 (±8.2) to 13.0 (±6.8) at 3 months of STN-DBS (pairwise comparisons, *p* < 0.001). At 12 months, it was 15.2 (±8.4) (*p* = 0.073). Analyses of each Scopa-Aut domain showed significant improvement at 12 months only for the thermoregulatory domain. Interestingly, from that domain, only excessive sweating during day and night improved significantly (sudomotor symptoms, question 17 (*p* = 0.001) and question 18 (*p* = 0.044).

### Regression analyses

Multivariable linear regression analyses were performed to identify predictive factors and covariables of the changes observed in PDSS and Scopa-Aut scores from baseline to 12 months of STN-DBS. Variables entered into the regression models were chosen based on clinical experience and exploratory correlation analyses. The results are shown in Table 3. For PDSS difference after 1 year of STN-DBS, the predictive model showed goodness of fit of only 1% (*p* = 0.468) and the model of covariance showed goodness of fit of 8% (*p* = 0.135). For Scopa-Aut difference, the predictive model showed goodness of fit of 3% (*p* = 0.590), whereas the covariance model showed goodness of fit of 31% (*p* < 0.001), with improvement in PDQ-39 as the only significant contributor. There is no violation of the assumption of normality, linearity, or multiple collinearity in the models. There are some overlapping questions in the questionnaires that might explain at least parts of the prediction of the variance, but Scopa-Aut and PDQ-39 only share the questions of feeling unpleasantly hot or cold.

**Table 3:** Multivariable linear regression analyses.

1. Prediction model of PDSS difference (from preop. to 12 months)		2. Covariance model of PDSS difference (from preop. to 12 months)	
Preoperative	*B*-coefficient (S.E.)	Difference from preoperative to 12 months	*B*-coefficient (S.E.)
Scopa-Aut	0.471 (0.381)	Scopa-Aut	−0.893 (0.481)
PDQ-39	−0.521 (0.325)	PDQ-39	−0.213 (0.312)
MDS-UPDRS II	0.331 (0.554)	MDS-UPDRS II	−0.574 (0.456)
MDS-UPDRS III off	−0.237 (0.238)	MDS-UPDRS III off	−0.095 (0.245)
LEDD	−0.237 (0.238)	LEDD	−0.005 (0.007)
Model fit:			
*R* square adjusted	1%		8%
(*p* value)	(0.468)		(0.135)

1. Prediction model of Scopa-Aut difference (from preop. to 12 months)		2. Covariance model of Scopa-Aut difference (from preop. to 12 months)	
Preoperative	*B*-coefficient (S.E.)	Difference from preoperative to 12 months	*B*-coefficient (S.E.)
PDSS	−0.021 (0.063)	PDSS	−0.081 (0.048)
PDQ-39	0.043 (0.121)	PDQ-39	0.308 (0.085)^a^
MDS-UPDRS I	−0.086 (0.205)	MDS-UPDRS I	0.145 (0.212)
MDS-UPDRS II	0.048 (0.170)	MDS-UPDRS II	−0.132 (0.154)
LEDD	0.003 (0.002)	LEDD	0.002 (0.002)
Model fit:			
*R* square adjusted	3%		31%
(*p* value)	(0.590)		(<0.001)^a^

Dependent variables are the differences in Parkinson’s Disease Sleep Scale (PDSS) and Scopa-Aut total scores from preoperative to 12 months of STN-DBS. As independent variables, preoperative scores are used in the prediction models (1, left column) and differences in scores from preoperative to 12 months in the covariance models (2, right column).

*PDQ-39* Parkinson’s Disease Questionnaire-39, *LEDD* Levodopa equivalent daily doses, *MDS-UPDRS* Movement Disorder Society revision of the Unified Parkinson’s Disease Rating Scale.

^a^Significant (*p* < 0.05).

## Discussion

In this prospective study of 60 PD patients treated with STN-DBS, our main finding was improvement of sleep disturbances at 12 months, as evaluated by PDSS. The domains overall sleep quality, nocturnal restlessness, nocturnal motor symptoms, and daytime dozing all improved. Statistically significant improvement in autonomic symptoms was also found at 3 months but was no longer evident at 12 months, except for sudomotor function.

Objective improvement in sleep quality following STN-DBS has been shown by polysomnography in small numbers of patients^32–34^, but there are conflicting results in the literature. Polysomnography cannot detect excessive daytime sleepiness and it is not feasible to study all PD patients with this modality. Therefore, several self-rating questionnaires that solely evaluate sleep have been developed, such as Epworth sleepiness scale, Pittsburgh Sleep Quality Index, and PDSS. Compound scales used to evaluate NMS also include sleep items (UPDRS I and II, NMSS, NMSQ). However, varying use of these scales has resulted in no uniform presentation of sleep symptoms. We have used the PDSS recommended by the MDS Task Force because it includes most of the potential sleep disturbances that PD patients may encounter^35^.

Overall, previous reports indicate that STN-DBS may improve nocturnal sleep in PD patients, particularly sleep quality^16^. However, many previous studies have included small numbers of patients followed for a short time period. In 10 patients with 3 months of follow-up, PDSS showed improved sleep quality but, unlike our findings, without improvement of excessive daytime sleepiness^36^. In two other studies using PDSS, one showed improved scores for daytime sleepiness, sleep quality, and restless legs syndrome (*n* = 17) at 6 months postoperatively^37^, and the other showed significant improvements in PDSS after 6 months (*n* = 40) but not after 12 months (*n* = 26)^38^.

Reduction of LEDD is hypothesized to play a role in the improvement of sleep symptoms, as studies have shown poorer sleep quality and less rapid eye movement (REM) sleep on higher doses of dopaminergic medication taken prior to sleep^39,40^. However, findings in a study that compared untreated PD patients, advanced PD patients (Hoehn & Yahr (H&Y) 4–5), and healthy controls indicated that sleep disruption occurs even before pharmacologic therapy and is likely a symptom of the underlying disease as well^41^. In a study comparing globus pallidus interna- and STN-DBS, there was no difference in PDSS improvements, despite a more pronounced LEDD reduction in the STN group. This may imply that the DBS effect on sleep surpasses the effect of dopaminergic medication reduction^42^.

Improvement in nocturnal mobility has also been suggested to be a significant contributor to improved sleep. However, Monaca et al. found only a mild motor improvement, but a significant improvement in sleep. They suggested that motor improvement alone could not fully explain sleep improvement, indicating that a possible direct effect on sleep/wakefulness regulatory centers might be involved^34^. A polysomnography study found that motor symptoms and medication explained only 10–30% of the variation of sleep efficiency, percentage of different sleep stages (including REM sleep), and total sleep time. These authors also suggest that other factors, related to changes in the disease process itself, are impacting sleep quality in PD^43^.

The fact that PD is perceived as a network disorder is contributing to the complexity of this field. The clinical effects of STN-DBS has been suggested to result from modulations of the connectivity within the cortico-striato-thalamo-cortical loop^44^ and of basal ganglia circuits that also affect sleep physiology^45^. Furthermore, the pedunculopontine nucleus and globus pallidus externa are both connected to STN^46–48^ and are important in sleep regulation. Studies have shown that DBS in these areas increases REM sleep^46,49^.

The causes of sleep disturbances thus seem complex and likely multi-factorial. Motor and non-motor PD symptoms, medication, dysregulation of sleep–wake function, and co-morbidities such as sleep-related breathing disorders can probably all play a role^50–52^. Therefore, the improvement of sleep quality probably also can be attributed to several mechanisms.

In this study, it was evident that both overall sleep quality and motor-associated sleep symptoms improved significantly. Sleep onset and maintenance insomnia improved less convincingly. Regression analyses did not reveal preoperative variables that predicted the PDSS reduction at 1 year or covariates to the PDSS improvement. Together, this may indicate that STN-DBS causes improvement in sleep disturbances through several mechanisms such as improvement in both nocturnal motor and NMS. There might also be a more direct effect on sleep–wake regulation centers.

Regarding autonomic symptoms, the significant improvement of Scopa-Aut total score at 3 months was no longer evident after 12 months of STN-DBS. However, thermoregulatory function remained significantly improved, due to reduced excessive sweating both during the day and night. This improvement of sudomotor function has been described in several previous publications^53^, and our results further confirm this.

Scopa-Aut total score has been reported to increase by age and disease duration^17^. In a study of 131 patients, Scopa-Aut scores worsened by 20% after 12 months of follow-up, and the worsening of autonomic symptoms correlated with reduced performance of daily activities of living and health-related quality of life^54^. Thus our findings of lower Scopa-Aut score at 12 months compared to the preoperative score (although not statistically significant) could still imply some degree of long-term improvement when accounting for the expected gradual worsening over time. Few previous reports have used Scopa-Aut to evaluate the effect of STN-DBS on autonomic symptoms. However, one study of 24 patients reported similar results as presented here, where Scopa-Aut initially improved but with subsequent deterioration^55^. Many studies focusing on NMS have used NMSS or NMSQ^56,57^, but these scales reflect total NMS burden and fluctuations and not autonomic dysfunction specifically. However, many of these studies show the same pattern of initial improvement of NMS with later worsening over time^23,25^.

We could not identify any preoperative factors that predicted the change in Scopa-Aut from preoperative to 12 months, but the parallel improvement of PDQ-39 was identified as a significant covariate. This might imply that improvements in autonomic symptoms contribute importantly to improved quality of life.

To our knowledge, this is the only long-term study that combines a relatively high number of patients, with specific evaluations of autonomic and sleep symptoms in PD patients after STN-DBS. The strengths of our study are the prospective design with systematic follow-up, the larger number of patients, and the longer follow-up time compared to previous studies. One limitation of our study is that the presented data were not planned as secondary outcomes but as preplanned investigations in a randomized study with motor symptoms and quality of life as primary endpoints. Thus, for the main variables presented in this article (PDSS and Scopa-Aut), the power to detect statistical differences between the two randomization groups of the parent study was not calculated. No such differences were found, and therefore we studied longitudinal changes of these variables in the whole study population. Multiple testing has been performed, but we have tried to correct for this using Bonferroni correction. No control group was included.

In summary, our study confirms a significant and lasting benefit of STN-DBS on sleep quality in PD patients with motor fluctuations. Some aspects of autonomic dysfunction also improved, although only thermoregulatory (sudomotor) function remained significantly improved at 12 months. We could not identify preoperative factors that predicted improvement in PDSS or Scopa-Aut. We did, however, find a close relationship between improved Scopa-Aut and improved quality of life at 1 year. Quality of sleep and autonomic symptoms deserve more focus both in the preoperative and postoperative evaluation of PD patients for STN-DBS. Future studies should consider including these factors among the main outcomes, especially in studies focusing on optimal electrode location, closed-loop DBS, and when exploring new targets.

## Methods

From 2009 to 2013, 60 patients referred for STN-DBS to the Department of Neurology, Oslo University Hospital were included in a single-center, prospective, randomized, double-blind study. We compared two methods of using up to five trajectories of preoperative microelectrode recordings (MER): single sequential versus multiple simultaneous introduction of MER to guide the placement of the permanent electrode. The detailed description of the study design (including surgical procedure) and the main results of this study on motor and quality of life outcomes have been published previously^29^. The primary endpoints of the parent trial were the differences in motor outcome (scores of MDS-UPDRS III medication-off) and quality of life (PDQ-39), from baseline to 1 year of STN-DBS. The multiple simultaneous MER group had a significantly greater improvement both in MDS-UPDRS III off score and in two PDQ-39 domains (activities of daily living and bodily discomfort)^29^.

Inclusion criteria for surgery were levodopa-responsive PD, with motor fluctuations including dyskinesia or medication-resistant tremor and/or intolerable side effects of dopaminergic drugs. Before inclusion, a neurologist, a psychiatrist, and a neuropsychologist evaluated all patients. Exclusion criteria included previous surgery for PD, marked axial motor symptoms, unresponsiveness to levodopa, significant cognitive impairment, major psychiatric disorders, or significant abnormalities on neuroimaging. All patients signed written informed consent. We have complied with all relevant ethical regulations, and the regional ethics committee (REK) approved the study.

### Neurologic evaluations

Patients were investigated with the MDS-UPDRS to assess non-motor experiences of daily living (Part I), motor experiences of daily living (Part II), motor examination (Part III), and the severity and impact of motor fluctuations (Part IV)^58^. The MDS-UPDRS III (range 0–132) was scored after overnight withdrawal of dopaminergic drugs (medication-off) and after a levodopa dose approximately 1.5 times the patient’s usual morning dose (medication-on). Postoperative evaluations were always made in the stimulation-on state. The H&Y score (0–5) was performed according to the recommendations of the MDS task force^59^. LEDD were calculated as advised by Tomlinson et al.^30^. Disease-specific health-related quality of life was assessed with PDQ-39^60,61^.

As part of the preplanned investigations performed at baseline, and after 3 and 12 months of STN-DBS, sleep disturbances were assessed with the PDSS and autonomic symptoms with the Scopa-Aut questionnaire. PDSS is a self-rated scale designed to measure common nocturnal problems, sleep disturbances, and excessive daytime sleepiness over the previous week^62^. It consists of 15 items to be scored from 0 (symptom severe and always present) to 10 (symptom-free), and maximum score is 150 (patient is free of all symptoms). Cutoff values of ≤83 for severe sleep abnormalities and ≤120 to detect sleep disturbances have been proposed^31,63^. The Scopa-Aut is a self-administered questionnaire, consisting of 26 items. It assesses the following domains: gastrointestinal symptoms (7 items), urinary symptoms (6 items), cardiovascular symptoms (3 items), thermoregulation (4 items), pupillomotor function (1 item), and sexual function (2 separate items for each gender)^64^. Each item is scored from 0 (never) to 3 (often), except for question 26, which is a yes/no question, and consequently not included in our statistical analysis. Total score ranges from 0 to 69 (for both genders), with higher scores expressing more severe symptoms.

### Statistical analysis

Because there were no significant differences in the changes of PDSS or Scopa-Aut from preoperative to 3 months and 12 months between the two randomization groups (independent sample *t* tests), we analyzed the two groups as one sample for the purpose of this paper.

The sample was assessed with tests for normality. Except for MDS-UPDRS IV at 3 and 12 months, variables were normally distributed. One-way repeated-measures ANOVA were performed to determine the difference between the preoperative and postoperative scores. For MDS-UPDRS IV, the Friedman Test was performed. Bonferroni corrections were made for PDSS with denominator 9 (significance level *p* < 0.006) and for Scopa-Aut denominator 7 (significance level *p* < 0.007). Where significant *p* values were found, pairwise comparisons were also performed to see if the significant change was for both time points. In SPSS, this is done in the same model and based on estimated marginal means. These *p* values are also Bonferroni corrected by multiplying for the three tests (*p* < 0.02).

We defined three subgroups of PDSS severity **(**PDSS ≤83 (indicating severe sleep abnormality), PDSS 84–120 (detecting sleep disturbances), and PDSS ≥121 (no sleep disturbance detected) according to proposed cutoff values. McNemar’s test was performed to compare whether the proportion of patients in these subgroups changed from preoperative to 3 months and from preoperative to 12 months.

Pearson’s rank order correlation was performed to compare scores of PDSS, Scopa-Aut, PDQ-39, MDS-UPDRS (I, II, III both medication-off and medication-on, VI), H&Y, LEDD, age at surgery, and duration of PD for both preoperative and 12-month follow-up scores and for the changes of scores between these time points. For correlation with MDS-UPDRS IV, we also used Spearman’s rank correlation, which showed similar correlation coefficients and *p* levels.

We performed multivariable linear regression analysis to assess (1) which *preoperative factors* might predict the changes in PDSS and Scopa-Aut from preoperative to 12 months of STN-DBS (prediction models) and (2) whether *changes in other selected variables* during this treatment period were associated with the changes observed in PDSS and Scopa-Aut scores (covariance models). The variables entered in the models were chosen based on clinical experience and knowledge, hypotheses presented in the literature on variables that can affect improvement in sleep and autonomic symptoms, and through exploratory correlation analyses. We included all variables that showed significant correlation (≤0.05, 2-tailed). We used tolerance (>0.10) and variance inflation factor (<10) to assess for multicollinearity and checked that correlation was <0.7. We used a forced entry method, which means that all predictor variables are tested in one block to assess their predictive ability while controlling for the effects of other predictors in the model. The model fit is presented by adjusted *R* square.

Missing single-item data points for PDSS, Scopa-Aut, and PDQ-39 (<1% of total items) were imputed using the last-observation carried-forward method. If the entire questionnaire was missing, these patients were excluded from the analysis. All statistical analyses were performed using IBM SPSS.22.

### Reporting summary

Further information on research design is available in the Nature Research Reporting Summary linked to this article.

## Supplementary information

Reporting Summary

## Data Availability

The data that support the findings of this study are not publicly available due to restrictions by Norwegian data protection regulations. The data will be available from the corresponding author upon reasonable request.
