# Supplementary material for: Subthalamic deep brain stimulation improves sleep and excessive sweating in Parkinson’s disease
Source: NPJ Parkinsons Dis. 2020 Oct 14;6:29. doi: 10.1038/s41531-020-00131-0 (PMC7560751; doi:10.1038/s41531-020-00131-0)
Supplement: Supplementary file 1 — Reporting Summary [file 41531_2020_131_MOESM1_ESM.pdf]

nature research

Corresponding author(s): NPJPARKD-00335R

Last updated by author(s): 2019.11.18

Reporting Summary

Nature Research wishes to improve the reproducibility of the work that we publish. This form provides structure for consistency and transparency in reporting. For further information on Nature Research policies, see Authors & Referees and the Editorial Policy Checklist.

Statistics

For all statistical analyses, confirm that the following items are present in the figure legend, table legend, main text, or Methods section.

n/a	Confirmed	
☐	☒	The exact sample size (n) for each experimental group/condition, given as a discrete number and unit of measurement
☐	☒	A statement on whether measurements were taken from distinct samples or whether the same sample was measured repeatedly
☐	☒	The statistical test(s) used AND whether they are one- or two-sided<br>*Only common tests should be described solely by name; describe more complex techniques in the Methods section.*
☐	☒	A description of all covariates tested
☐	☒	A description of any assumptions or corrections, such as tests of normality and adjustment for multiple comparisons
☐	☒	A full description of the statistical parameters including central tendency (e.g. means) or other basic estimates (e.g. regression coefficient) AND variation (e.g. standard deviation) or associated estimates of uncertainty (e.g. confidence intervals)
☐	☒	For null hypothesis testing, the test statistic (e.g. F, t, r) with confidence intervals, effect sizes, degrees of freedom and P value noted<br>*Give P values as exact values whenever suitable.*
☒	☐	For Bayesian analysis, information on the choice of priors and Markov chain Monte Carlo settings
☒	☐	For hierarchical and complex designs, identification of the appropriate level for tests and full reporting of outcomes
☒	☐	Estimates of effect sizes (e.g. Cohen's d, Pearson's r), indicating how they were calculated

Our web collection on statistics for biologists contains articles on many of the points above.

Software and code

Policy information about availability of computer code

Data collection	No software was used.
Data analysis	SPSS 22.

For manuscripts utilizing custom algorithms or software that are central to the research but not yet described in published literature, software must be made available to editors/reviewers. We strongly encourage code deposition in a community repository (e.g. GitHub). See the Nature Research guidelines for submitting code & software for further information.

Data

Policy information about availability of data

All manuscripts must include a data availability statement. This statement should provide the following information, where applicable:

- Accession codes, unique identifiers, or web links for publicly available datasets
- A list of figures that have associated raw data
- A description of any restrictions on data availability

The data that support the findings of this study are not publicly available due to restrictions by Norwegian data protection regulations. The data will be available from the corresponding author upon reasonable request.

Field-specific reporting

Please select the one below that is the best fit for your research. If you are not sure, read the appropriate sections before making your selection.

☒ Life sciences ☐ Behavioural & social sciences ☐ Ecological, evolutionary & environmental sciences

Life sciences study design

All studies must disclose on these points even when the disclosure is negative.

Sample size	Sample size calculations were reported for the parent study ref. article Bjerknes et al"Multiple Microelectrode Recordings in STNDBS Surgery for Parkinson's Disease: A Randomized Study". Movement Disorders Clinical Practice, 2008.
Data exclusions	Between April 2009 and December 2013, 76 patients underwent STN-DBS surgery at Oslo University Hospital. Sixty patients were included in the study. 7 declined to partcipate, 4 had language barrier, 1 had psychiatry, 1 had chronic hip infection, 3 were not asked.
Replication	Not applicable.
Randomization	Patients were randomly assigned in 1:1 ratio to the sMER or mMER groups in blocks of 4 or 6, by a computerized randomization generator handled by the Office of Clinical Research, an independent body at Oslo University Hospital.
Blinding	Both the patients and the neurologists performing post-operative assessments remained blinded.

Reporting for specific materials, systems and methods

We require information from authors about some types of materials, experimental systems and methods used in many studies. Here, indicate whether each material, system or method listed is relevant to your study. If you are not sure if a list item applies to your research, read the appropriate section before selecting a response.

Materials & experimental systems

n/a	Involved in the study	
☒	☐	Antibodies
☒	☐	Eukaryotic cell lines
☒	☐	Palaeontology
☒	☐	Animals and other organisms
☐	☒	Human research participants
☐	☒	Clinical data

Methods

n/a	Involved in the study	
☒	☐	ChIP-seq
☒	☐	Flow cytometry
☒	☐	MRI-based neuroimaging

Human research participants

Policy information about studies involving human research participants

Population characteristics	Baseline charecteristics for age at surgery, gender, duration of disease at surgery, HAD, Mattis DRS, MDS-UPDRS I-IV, HY and LEDD for the whole population is included in the article.
Recruitment	Between April 2009 and December 2013, 76 patients underwent STN-DBS surgery at Oslo University Hospital. Sixty patients (15 women, 45 men) participated in the study. Not included: 7 patients declined to partcipate, 4 were not included due to language barrier, one had psychiatric manifestations, one had chronic hip infection and three were not asked to partcipate.
Ethics oversight	Regional ethics committee (REK).

Note that full information on the approval of the study protocol must also be provided in the manuscript.

Clinical data

Policy information about clinical studies

All manuscripts should comply with the ICMJE guidelines for publication of clinical research and a completed CONSORT checklist must be included with all submissions.

Clinical trial registration	ClinicalTrials.gov Identifier: NCT00855621
Study protocol	Will be available from the corresponding author upon request.
Data collection	From 2009 to 2013, 60 patients referred for STN-DBS to the Department of Neurology, Oslo University Hospital, were included in a single-center randomized prospective study with double-blind comparison of the use of single sequential versus multiple simultaneous microelectrode-recordings to guide the placement of the permanent electrode. Patients were examined at the Dept of Neurology by a Neurologist specializing in movement disorders. Evaluations were performed preoperatively, 3 months and 12 months postoperatively.
Outcomes	The primary endpoints of the parent trial were the differences in scores from baseline to 1 year of STN-DBS, of MDS-UPDRS III medication-off and PDQ-39.
